# Next Generation Immunotherapy for Pancreatic Cancer: DNA Vaccination is Seeking New Combo Partners

**DOI:** 10.3390/cancers10020051

**Published:** 2018-02-16

**Authors:** Paola Cappello, Claudia Curcio, Giorgia Mandili, Cecilia Roux, Sara Bulfamante, Francesco Novelli

**Affiliations:** 1 Department of Molecular Biotechnology and Health Sciences, University of Turin, Turin 10126, Italy; paola.cappello@unito.it (P.C.); claudia.curcio@unito.it (C.C.); giorgia.mandili@unito.it (G.M.); cecilia.roux@unito.it (C.R.); sara.bulfamante@unito.it (S.B.); 2 Molecular Biotechnology Center (MBC), University of Turin, Turin 10126, Italy; 3 Center for Experimental Research and Medicine Studies (CERMS), Azienda Ospedaliera Città della Salute e della Scienza di Torino, Turin 10126, Italy

**Keywords:** pancreatic ductal adenocarcinoma, alpha-enolase, DNA vaccination, immunotherapy, PI3K inhibitors, tumor-associated macrophages, chemotherapy

## Abstract

Pancreatic Ductal Adenocarcinoma (PDA) is an almost incurable radio- and chemo-resistant tumor, and its microenvironment is characterized by a strong desmoplastic reaction associated with a significant infiltration of T regulatory lymphocytes and myeloid-derived suppressor cells (Tregs, MDSC). Investigating immunological targets has identified a number of metabolic and cytoskeletal related molecules, which are typically recognized by circulating antibodies. Among these molecules we have investigated alpha-enolase (ENO1), a glycolytic enzyme that also acts a plasminogen receptor. ENO1 is also recognized by T cells in PDA patients, so we developed a DNA vaccine that targets ENO1. This efficiently induces many immunological processes (antibody formation and complement-dependent cytotoxicity (CDC)-mediated tumor killing, infiltration of effector T cells, reduction of infiltration of myeloid and Treg suppressor cells), which significantly increase the survival of genetically engineered mice that spontaneously develop pancreatic cancer. Although promising, the ENO1 DNA vaccine does not completely eradicate the tumor, which, after an initial growth inhibition, returns to proliferate again, especially when Tregs and MDSC ensue in the tumor mass. This led us to develop possible strategies for combinatorial treatments aimed to broaden and sustain the antitumor immune response elicited by DNA vaccination. Based on the data we have obtained in recent years, this review will discuss the biological bases of possible combinatorial treatments (chemotherapy, PI3K inhibitors, tumor-associated macrophages, ENO1 inhibitors) that could be effective in amplifying the response induced by the immune vaccination in PDA.

## 1. Self-Antigens Acting as Tumor-Associated Antigens (TAAs) Are Recognized by Antibodies in PDA

The immunosurveillance theory, which establishes the ability of the immune system to recognize and hinder the progression of a tumor, is more than a century old [1]. It has been ascertained that only an in-depth knowledge of the various immune populations and of the mechanisms regulating their functions has allowed this theory to be refined, leading to the well-known theory of “immunoediting” [2]. Based on the idea of exploiting the immune system to directly fight tumor progression, immunotherapy has thus been developed. The crucial point of effective immunotherapy is to identify the best “tumor-associated target” and combine specific activation of the adaptive immune response with the defined tumor target, including strategies focused on the release from their natural brakes (immune checkpoints), ensuring a minimal risk of eliciting autoimmunity, or limiting immunosuppressive mechanisms.

For many years, our group has studied the relationship between tumors and the immune system, in particularly in pancreatic ductal adenocarcinoma (PDA). It is well known that an inflammation-associated desmoplastic reaction, typical of this kind of tumor, creates an immune-deviated suppressive microenvironment that favors cancer progression in place of an effective antitumor effector response [3]. In the last 10 years, we have discovered and characterized the antibody response in PDA patients, and we have demonstrated the efficacy of the autoantibodies and related antigens as diagnostic markers and therapeutic targets. The autoantibody response of PDA patients reflects the complex interplay between the microenvironment and the tumor: most of the identified targets are metabolic and cytoskeleton molecules whose expression is deregulated in PDA, which heavily influence the overgrowth of PDA and its ability to disseminate through the extracellular matrix, and to rewire its metabolic pathway to fuel proliferation and evade immune system patrolling.

In our first study published in 2007, we demonstrated the presence of autoantibodies in the sera of PDA patients that could discriminate them from healthy subjects and patients with chronic pancreatitis or other malignancies [4]. Sera from PDA patients, healthy subjects, patients with non-PDA cancers and chronic pancreatitis patients were analyzed, and autoantibodies and the relative antigens were identified using a SERological Proteome Analysis (SERPA) approach. The proteomes of three human pancreatic tumor cell lines (CFPAC-1, MiaPaCa-2, and BxPC-3) were separated by two-dimensional-electrophoresis (2-DE), and electro-transferred onto a nitrocellulose membrane. The obtained maps were stained with sera, and the spots recognized by antibodies were identified by mass spectrometry. By comparing the 2-DE maps of the four groups (PDA, healthy subjects, other malignancies and chronic pancreatitis patient sera), only nine proteins were recognized by PDA patient antibodies, namely triosephosphateisomerase 1 (TPIS), retinal dehydrogenase 1 (AL1A1), glucose-6-phosphate 1-dehydrogenase (G6PD), elongation Factor Tu (EFTU), isocitrate dehydrogenase (IDHC), keratin 10 (K1C10), cofilin-1 (COF1), transgelin (TAGL) and alpha-enolase (ENO1). Most of these proteins have been demonstrated to be up-regulated in tumors. As these antigens are self-proteins, the antibody response against them could be explained as the result of breaking self-tolerance [4].

We focused on ENO1, a glycolytic enzyme that catalyzes the conversion of 2-phosphoglycerate to phosphoenolpyruvate, but also acts as a plasminogen receptor. ENO1 is over-expressed in many cancers, including pancreatic cancer [5,6,7,8,9,10]. Notably, we found that ENO1 induced a high frequency of antibody responses in PDA patients [4]. However, a more specific antibody response to ENO1 in PDA patients was observed against its phosphorylated isoforms [6]. In a second SERPA study, when sera from PDA, non-PDA cancer, chronic pancreatitis, autoimmune disease patients and healthy subjects were compared in terms of antibody reactivity, six isoforms of ENO1 with the same molecular weight but different isoelectric points, were identified [6]. Four isoforms out of these six were recognized by almost all sera, while the two most acidic isoforms were recognized by over 60% of PDA but by only 4% of non-PDA and 9% of chronic pancreatitis patient sera, suggesting a role as a PDA biomarker. This hypothesis was confirmed by the presence of these two isoforms in PDA, but not in normal pancreatic tissue [6]. Mass spectrometry analysis revealed phosphorylation on serine 419 of these two isoforms [11], and their role as biomarkers was confirmed by statistical analysis. Indeed, autoantibodies against the two isoforms discriminated PDA patients from controls with 62% sensitivity and 97% specificity, and combined with the tumor marker CA 19.9, they were able to ameliorate diagnostic performance. This could be further improved by combining with autoantibodies against Ezrin, another antigen identified in the sera of the same cohort of patients [12]. Indeed, a diagnostic algorithm that considered Ezrin-autoantibodies and CA 19.9, and in the discordant cases, the presence or absence of ENOA1,2-autoantibodies, had 100% sensitivity and 92.3% specificity [12]. Autoantibodies against Ezrin appeared to be particularly important as diagnostic tools, because their presence has been demonstrated in a pre-diagnostic cohort of patients and at the early stage of disease in two genetically engineered mouse models (GEM) of PDA [12]. Interestingly, the presence of autoantibodies against phosphorylated isoforms of ENO1, but not Ezrin, correlated with a better prognosis in advanced PDA patients [12]. In addition to a humoral response, ENO1 is also able to elicit a cellular response in PDA patients [5]. ENO1 was able to elicit both CD4 and CD8 T cell proliferation and IFNγ production. Importantly, ENO1 induced antitumor cytotoxic effector T cells without affecting normal cells [5]. All these data demonstrated that autoantibody characterization may lead to identifying hits, such as ENO1 and Ezrin, which represent promising therapeutic targets in PDA. In addition, the presence of autoantibodies to ENO1 in other cancer patients as mentioned before, renders ENO1 a good molecular candidate target in other types of cancers too.

## 2. ENO1 as a Target for PDA Immunotherapy

Over the last decade, great efforts have been invested in developing approaches for eliciting anti-tumor responses by priming a novel (or boosting an existing) immune response against tumor cells. These have included different strategies from antibodies to vaccines, and the huge amount of pre-clinical and clinical results have led to the approval of some of these treatments by the U.S. Food and Drug Administration agency and the European Medicines Agency, as immunotherapy for cancer patients.

Although immunotherapy has been widely explored for cancer treatment, PDA seems to be unsuitable for this approach as it is considered an “immune privileged site”. This is due to a low rate of mutations that generate neo-antigens [13], together with an immunosuppressive environment. However, we have demonstrated the presence of anti-ENO1 autoantibodies in PDA patient sera [6] and of anti-ENO1-specific T cells into the tumor [5,14,15]. By cloning tumor-infiltrating lymphocytes (TILs) from both marginal and center tumor tissues of surgically resected PDA patients, we clearly obtained a different set of ENO1-specific T cells: most patients displayed a higher number of clones with a Th1/Th17 (IFNγ and/or IL17 producers) phenotype in the marginal tumor area paralleled by a higher number of clones with a T regulatory lymphocytes (Treg) phenotype (FoxP3^+^ and IL10 producers) [14]. These results suggested the presence of antigen-specific T cells into the tumor that, unfortunately, are frustrated in their functions by the presence of Tregs. However, analyzing peripheral T cell clones from the same patients revealed that those having more peripheral ENO1-specific T clones were also surviving longer [15]. Therefore, the presence of anti-ENO autoantibodies and T cells prompted us to verify the hypothesis of eliciting a strong anti-ENO1 immune response by a DNA vaccine, able to counteract tumor progression.

**Figure 1:**
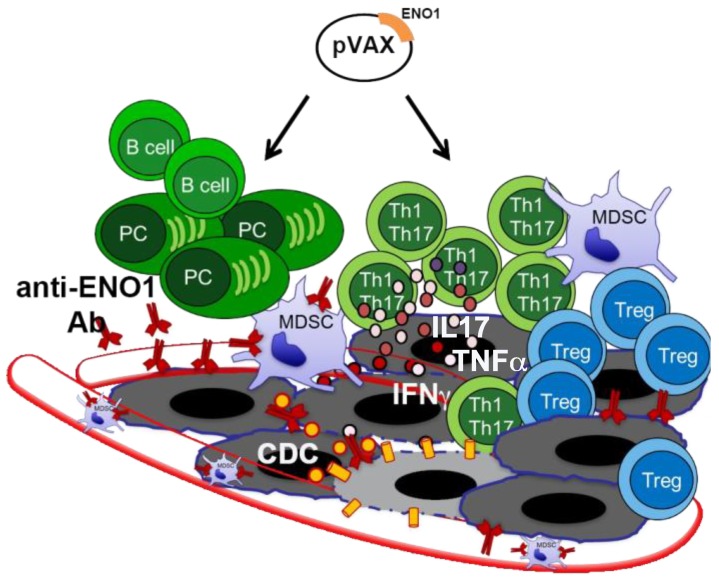
Alpha-enolase (ENO1) DNA vaccination effects in pancreatic ductal adenocarcinoma (PDA) mouse model. Cartoon shows the multiple effects of ENO1 DNA vaccination on the antitumor immune response (black arrows): activation of B cells producing anti-ENO1 antibodies (Ab) that affect tumor cells and myeloid-derived suppressor cells (MDSCs) invasion and endothelial adhesion (vessels are shown as transparent red tubes). Moreover, vaccine induces complement-dependent cytotoxicity (CDC) of tumor cells (grey cells) and T cells, specially Th1/Th17 cells that release IL17, TNFα and IFNγ cytokines. Yellow circles and cylinders indicate the complement system and the membrane attack complex, respectively, involved in the CDC. Circles represent cytokines; plasma cell (PC).

To do this, we exploited a sophisticated GEM model that spontaneously develops PDA due to the pancreas-specific expression of a Cre recombinase that cuts off a STOP cassette before the mutated Kras and/or TP53 genes [16]. Based on the expression of mutated Kras alone, or in combination with mutated TP53, mice are called KC or KPC. In our setting, KC and KPC mice were vaccinated when Pancreatic Intraepithelial Neoplasia (PanINs) lesions were already present, and they received a total of three (KC) and four (KPC) rounds of immunization every 3 and 2 weeks, respectively. The ENO1 vaccine induces a specific integrated humoral and cellular response that efficiently prolonged mouse survival from 10% to 32% in the KPC and KC mice, respectively [17]. A therapeutic setting of ENO1-DNA vaccine was also able to significantly decrease the size of well-established in situ adenocarcinomas. Several mechanisms were demonstrated to be responsible for this effect: the induction of anti-ENO1 antibodies, which mediated complement-dependent cytotoxicity, inhibited tumor cell invasion [18,19] and myeloid-derived suppressor cell (MDSC) infiltration into the tumor [20]; and the expansion of Th1 and Th17 cells, which contributed—with their cytokine—to inhibit tumor cell growth and to elicit the B cell-isotype switch. The ENO1 DNA vaccine also significantly decreased Treg infiltration into the tumor area and increased infiltration of effector CD3 cells (Figure 1) [17].

The crucial role of anti-ENO1 antibodies was confirmed by the observation that ENO1 vaccinated mice showed B cells organized in dense aggregates that displayed a distinct structure, the so-called tertiary lymphoid tissue (TLT), which were not found in normal pancreases, and only sporadically in PDA of untreated mice or those vaccinated with an empty-vector [21]. B cells organized into TLT, namely CD20-TLT, were shown to correlate with a better prognosis and with a greater infiltration of CD8^+^ T cells in a cohort of 104 PDA patients. Mice orthotopically injected with syngeneic PDA cells, in which no TLT was observed compared to the GEM, and depleted of B cells by a single injection of an anti-CD20 Ab, displayed a dramatic reduction of circulating B cells as well as CD20-TILs. The anti-CD20 treatment induced a significant increase in genes related to T and NK cell recruitment, as well as genes involved in lymphoid tissue structure development and CD8^+^ T cell differentiation and maintenance. These results highlighted B cells as an essential element of the PDA microenvironment, and identified their spatial organization as a key regulator of their antitumor function [21].

Finally, as ENO1 overexpression occurs in almost all PDA cases, and the immune response to it is a common feature of PDA patients, the use of the whole ENO1 sequence like in our strategy, is potentially applicable to all patients without the need of personalizing therapy. ENO1, indeed, is different from the “neo-antigens” that represent individual tumor mutations and that require a personalized immunotherapy approach. This would also be an economic advantage.

## 3. Novel Therapeutic Combinations with Vaccination

As recent studies have demonstrated, targeting a single TAA does not appear to effectively treat tumors. However, the ENO1 DNA vaccine gave promising results and created the possibility of novel combinations in terms of including other TAAs (in multiple antigen vaccines) or strategies to improve the efficacy of the immune system “educated” by the vaccination approach. Accumulating evidence indicates that multiple anticancer agents, including classic chemotherapeutics as well as targeted compounds, stimulate tumor-specific immune responses either by inducing immunogenic cell death or by engaging immune effector mechanisms [22].

In the following part of the review, we will discuss different combinatorial therapeutic strategies to render the DNA vaccination approach more efficacious and long-lasting.

### 3.1. Exploiting Chemotherapy (CTX) Regimens to Increase the Effectiveness of ENO1 DNA Vaccination

PDA remains very challenging to treat, with a cure rate of just 7%. The gold standard cure is surgical resection, which can, unfortunately, only be performed in 20% of patients [23]. Two effective CTX regimens-gemcitabine/nab-paclitaxel and Folfirinox (a mixture of oxaliplatin, irinotecan, folinic acid and 5-fluorouracil) have led to improved outcomes in metastatic patients, and also represent attractive neoadjuvant treatment strategies for locally advanced disease [24]. However, significant differences in outcomes cannot be achieved without novel strategies.

**Figure 2:**
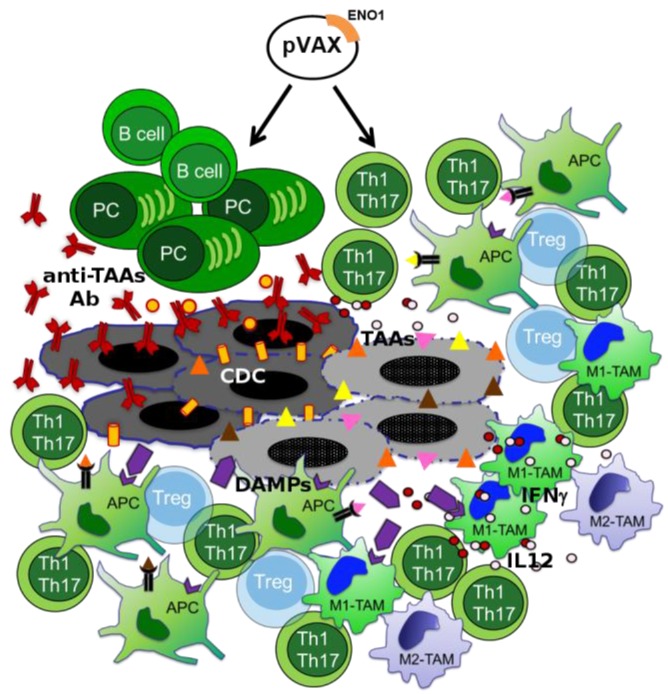
Effects of the ENO1 DNA vaccination and chemotherapy combination. Multiple effects of ENO1 DNA vaccination and chemotherapy (CTX) on innate and adaptive anti-tumor responses are shown. Transparent Tregs represent inhibited cells; triangles, TAAs; violet symbols, damage-associated molecular patterns (DAMPs) and DAMP receptors; antigen presenting cell (APC); tumor associated macrophage (TAM); M1-like phenotype TAM (M1-TAM); M2-like phenotype TAM (M2-TAM).

In the last 10 years, the capacity of CTX to elicit an antitumor immune response has acquired new interest. CTX affects cancer cells through several mechanisms that generally impair cell replication, such as DNA damage; thus, the consequent cellular stress results in cell death [25]. In addition to the two typical processes of cell death-necrosis and apoptosis some CTX agents induce an immunogenic cell death in which cancer cells express damage-associated molecular patterns (DAMPs), which are detected by receptors on a variety of innate immune cells, such as macrophages and neutrophils, but also on antigen presenting cells (APCs) (Figure 2) [26].

PDA displays an intense desmoplastic reaction characterized by a dense network of elements, including fibroblasts, immune cells and extracellular matrix (ECM), which together are active components of the tumor tissue. Furthermore, considering the immune modulating effects of some chemotherapeutic agents used in clinical practice [27,28], the combination of CTX and DNA vaccination could potentially increase their therapeutic efficacy.

Recent studies have suggested that neoadjuvant regimens could be immunologically more relevant than adjuvant treatments, as this therapeutic strategy minimizes the negative impact of tumor bulk on the potency of the antitumor immune response, and also allows CTX to modulate the immune phenotype of residual tumor cells [29]. The limited success achieved by previous studies on neoadjuvant therapy could be attributed to the choice of relatively less active regimens (with a tumor response rate of less than 20%), but novel polyCTX regimens are significantly more effective [30,31,32], suggesting their use in the perioperative setting as well.

CTX also seems to interfere with the mechanisms of tumor-induced immunosuppression. Low doses of CTX decreased the number of Tregs, along with their suppressive function, in rats bearing an established subcutaneous tumor from colon carcinoma [33]. These selective effects could be due to the permanent tumor-induced proliferation of Tregs, which makes them more sensitive to CTX, or their constitutive Foxp3 expression, which increases the production of proapoptotic molecules (Figure 2) [33].

Several studies have also demonstrated the positive effect of CTX on the antitumor immune response in PDA. In peripheral blood of advanced PDA patients, gemcitabine treatment induced an increase of the number and percentage of CD14^+^ monocytes and myeloid dendritic cells (DCs) [34]. In PDA resectable patients, tumor associated macrophages (TAMs) showed a predominant M2-like immunosuppressive phenotype (M2-TAM), and their presence at the stroma-tumor interface was correlated to a worse prognosis, with the exception of those patients who had undergone adjuvant CTX, as TAM density at the stroma-tumor interface was associated with a better prognosis compared to surgical resected patients [35]. Moreover, CTX modulated the interaction between macrophages and PDA cells in vitro, since gemcitabine synergized with the cytotoxic effect of M1-polarized macrophages (M1-TAM) and inhibited the pro-tumor effect of M2-TAM. This was due in part to the direct effect of gemcitabine on macrophages, which showed an increase of M1-like markers, such as IL-12 and IFNγ, and to the downregulation of M2-like markers, such as IL-10 (Figure 2) [35].

In an orthotopic mouse model of PDA, treatment with 5-FU combined with IFNα gave rise to a greater number of NK cells infiltrating the tumor [36]. Furthermore, NK cells isolated from these tumors showed a higher in vitro cytotoxicity against PDA cell lines, which in turn expressed higher levels of MHC-I molecules and NKG2D ligands, suggesting that CTX could have a potential role in eliciting the immunogenity of cancer cells [36].

Konduri et al. combined gemcitabine treatment with a DC-based vaccine leading to the elimination of metastasis and recurrence, and increasing the overall survival in an orthotopic mouse model of PDA. Mice treated with the combined therapy exhibited higher levels of effector CD8^+^IFNγ^+^CCR7^+^NK1.1^+^ T-cells in peripheral blood and, conversely, exhausted GITR^+^CD8^+^ T-cells were decreased. Moreover, retro-orbital tumor re-challenge of surviving animals demonstrated that only the mice that had received the combination therapy maintained the antitumor immunity post-treatment [37].

Immunogenicity owing to CTX is based not only on the activation of the innate immune system, the inhibition of Treg cell immunosuppression and the enhanced activation and ability of APCs in presenting the antigens, but also on the potential antigenicity of target cancer cells [26]. During tumorigenesis, cancer cells accumulate a series of mutations that can be recognized as non-self by the adoptive immune system.

However, the role of CTX in promoting the formation of neoantigens or in the modification of TAA expression levels has yet to be explored. For this reason, we are investigating—in PDA patients—the potential effect of CTX in inducing novel TAAs or in enhancing the antigenicity of the already established TAAs, such as ENO1, to enhance the positive effect of DNA vaccination with the combination of CTX treatment. Of note, to confirm the feasibility of this approach, we observed that sera from PDA patients treated with CTX displayed an increased frequency of antibodies (IgG) that recognized several TAAs, including ENO1, which are up-regulated in PDA [38]. Interestingly, after CTX there was a positive correlation between the increased TAA-antibody recognition and better survival [38]. Notably, when the recombinant form of identified TAAs was used to stimulate autologous peripheral T cells in vitro from PDA patients before and after CTX, an increased T cell response was observed in PDA patients after CTX treatment [38]. This data demonstrated that the analysis of the PDA patient antibody response before and after CTX treatment was able to identify TAAs suitable for widening the spectrum of anti-tumor immunity achievable by vaccination in conjunction with the CTX treatment. This approach has been confirmed in preclinical studies in which we have observed that the combination of CTX and ENO1 vaccination in GEM mice was much more efficacious in inducing anti-ENO1 antibodies that ENO1 vaccination alone [38]. Our working hypothesis in the future is that PDA therapies can be implemented by targeting tumor stroma and immune infiltrating cells by selecting CTX strategies to boost the immune response (Figure 2).

### 3.2. Phosphoinositide 3-Kinase (PI3K) Inhibitors to Block Myeloid-Derived Suppressor Cells

PI3K regulates different pathways involved in cell survival, apoptosis, senescence, DNA repair, angiogenesis, cellular metabolism, motility, proliferation and differentiation, and has a key role in tumorigenesis [39,40,41,42,43,44,45] The PI3K lipid kinase family is divided into three classes according to their structure and substrate specificity. Class I PI3Ks are heterodimers formed by a regulatory and a catalytic (p110) subunit; these are further subdivided into class IA (PI3Kα, PI3Kβ, PI3Kδ) and IB (PI3Kγ), depending on the type of regulatory subunit in the complex (p85 or p84/p101, respectively) [45]. In the absence of activation signals, the catalytic subunit interacts with the regulatory subunit and inhibits kinase activity, while in the presence of a specific molecule (e.g., chemokine, growth factor, cytokine), which binds the tyrosine kinase receptor (RTK) or G protein-coupled receptor (GPCR), PI3Ks are recruited to the membrane where p110 is exposed, and PIP_2_ is phosphorylated into PIP_3_, leading to activation of AKT, and the regulation of different biological functions. Moreover, while class IA PI3Kα and β isoforms are widely expressed in endothelial, epithelial and tumor cells, PI3Kδ is expressed in T and B lymphocytes, and the class IB isoform PI3Kγ is expressed in leukocytes and especially in myeloid cells, where it is the major PI3K isoform [46,47,48].

Recently, PI3K inhibitors are being used in a clinical setting, and the number of scientists involved in this area has vastly expanded; the key discoveries that led to the molecular understanding of PI3K signaling and function will, therefore, be discussed [48]. Notably, the growth and metastatic spread of different types of transplanted tumors (i.e., melanoma, lung carcinoma and thymoma) in mice in which PI3Kδ was genetically inactivated, were significantly inhibited compared to those in normal mice [49]. In addition, PI3Kδ activity has been shown to be required for the proliferation and differentiation of suppressive inducible Treg cells, and its specific deletion in Treg cells delayed tumor growth and prolonged the survival of mice after tumor cell challenge [49,50]. The effectiveness of pharmacological inhibition of PI3Kδ was assessed in therapeutic conditions using a GEM model, namely KPC. This treatment prolonged survival and reduced the incidence of metastases and other disease-associated pathologies [49]. The relative abundance of peripheral Tregs in lymph nodes was reduced after 7 days of treatment, correlating with higher levels of CD44^high^CD8^+^ lymphocytes in the draining lymph nodes and relatively more infiltrating CD8^+^ T cells in pancreatic lesions at 14 days after treatment [49]. These data indicated that therapeutic targeting of p110δ can promote immune-mediated elimination of cancer [49,50].

**Figure 3:**
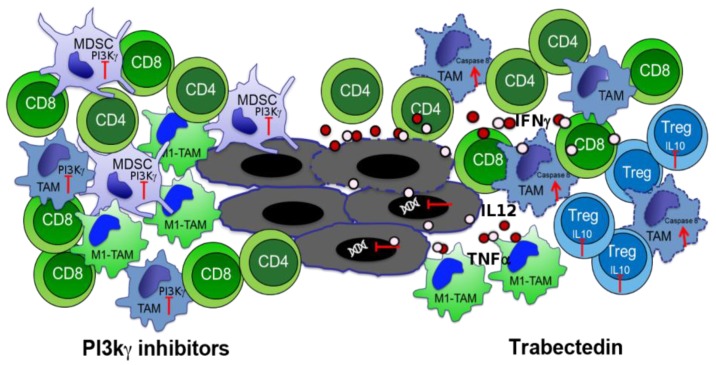
Phosphoinositide 3-Kinaseγ (PI3Kγ) inhibitor (**left**) and Trabectedin (**right**) effects on immune cells. CD8 recruitment dependent from M1-TAM switch of TAM and MDSC after PI3Kγ inhibition is represented. Caspase-8 activation and cytokine production induced by Trabectedin in TAM, IFNγ production by T cells and IL10 inhibition in Treg are shown.

PI3Kγ is expressed in human and murine tumor-associated macrophages and myeloid cells that are responsible for the increase in a suppressive microenvironment and fibrotic reaction into the tumor [51]. We have demonstrated that the selective genetic deletion and pharmacological inhibition of this kinase significantly impaired the orthotopic and spontaneous PDA tumor growth and metastasis by affecting myeloid cell functions [46]. In another study, it has been shown that both human and murine PDAs exhibited increased PI3Kγ-dependent Bruton tyrosine kinase (BTK) activation in CD11b^+^/Fcγ II/III^+^ myeloid cells, and that PI3Kγ inhibition, alone or in combination with gemcitabine, slowed the progression of orthotopic tumors [52]. Tumor suppression and increased mouse survival induced by PI3Kγ inhibition has been directly associated with the activation of CD8^+^ T cells and to M2-TAM switch into a more anti-tumoral M1-TAM phenotype (Figure 3) [46].

Notably, elective pharmacologic targeting of PI3Kγ restores sensitivity to immune checkpoint blockade. We demonstrated that targeting PI3Kγ, with a selective inhibitor, currently being evaluated in a phase 1 clinical trial (NCT02637531), can reshape the tumor immune microenvironment and promote cytotoxic T cell-mediated tumor regression, without targeting cancer cells directly [53].

Tumor stromal cells such as pancreatic stellate cells (PSCs) and immune cells create a microenvironment that protects cancer cells through a complex interaction, ultimately facilitating their local proliferation and their migration to different sites [54]. Activated PSCs play a pivotal role in the development of pancreatic fibrosis, thanks to the ability of actively proliferating, migrating, and producing ECM components, such as type I collagen, and expressing cytokines and chemokines [55]. Activation of PSCs is regulated by different key mediators of stimulatory and inhibitory signals (i.e., peroxisome proliferator-activated receptor-c, Rho/Rho kinase, NF-κB), mitogen-activated protein kinases, PI3K, Sma- and Mad-related proteins, and reactive oxygen species, the targeting of which could be of interest for developing anti-fibrosis therapy in the future [54,55,56]. It is very important to demonstrate that pharmacological inhibition of PI3Kγ could also affect PSCs.

**Figure 4:**
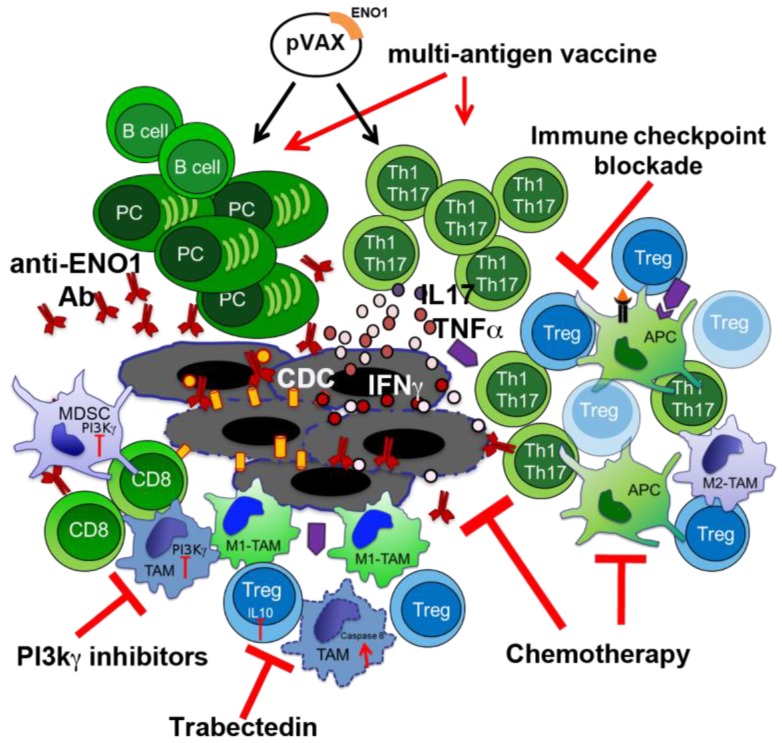
ENO1 DNA vaccination and potential combo partners. Cartoon shows the multiple effects of potential combinatory treatments (red arrows) with ENO1 DNA vaccination (black arrows) on the antitumor immune response.

Developing strategies focused on the inhibition of myeloid cell-mediated immune suppression, such as the use of checkpoint and/or other inhibitors can be of interest. All anti-tumor restoring effects of PI3K inhibitors strongly suggest that small pharmacological inhibitors that target PI3Kγ or δ isoforms or all isoforms together can be a suitable powerful combinatorial partner to enhance the antitumor efficacy of ENO1 vaccination (Figure 4). Despite only 20% of PDA patients displaying an increased activation of AKT/mTOR in tumor cells [57], the cited inhibitors directly impact leukocytes, and myeloid or Treg cells in particular. Although PI3K inhibitors down-regulate AKT activation and influence the regulation of downstream genes, included glycolytic enzymes as ENO1 [58], this will not affect tumor cells. Therefore, there are no potential restrictions for treatable patients.

### 3.3. Macrophage Targeting to Redirect Epigenetic Changes in Tumor Infiltrating Lymphocytes

It is well known that the presence of TILs is usually associated with a better prognosis [59]. In PDA as well, the elevated number of both infiltrating CD4 and CD8 T cells was demonstrated to correlate with a better outcome [60]. In previous studies, we have demonstrated the presence of T cells specific for the PDA-associated antigen ENO1, both in the tumor and in the blood of PDA patients [14,15,61]. These ENO1-specific TILs were frustrated in their Th1 and Th17 effector functions by ENO1-specific Tregs, and were much more representative in the marginal area than within the tumor, where Tregs were more numerous [14].

Not only Tregs but also TAMs or MDSC affect the status of T cells in cancer [62]. These populations are known to create an immune suppressive environment through either secretion of cytokines, such as IL10 and TGFβ, or expression of inhibitory molecules, such as PD-L1 [63,64], which inhibits the activation of CD8 T cells, and induces a switch of CD4 T cells towards Th2 and Treg phenotypes [65,66]. However, the presence of mixed stimuli in the microenvironment creates conditions for reversible changes in infiltrating cells, including TILs. These modifications derive from the activation or inhibition of signaling pathways, along with chromatin remodeling, which is highly involved in gene transcription control. In a recent study, we compared the epigenetic profile of infiltrating T cells in both normal and tumoral pancreata, with or without perturbation of the tumor stroma by depleting macrophages. To this end we used Trabectedin (Yondelis™), a sponge-derived drug that binds to the minor groove of DNA, causing blocking of proliferating cells, and interfering with transcription regulation and different DNA repair pathways (Figure 3) [67,68]. Trabectedin has been demonstrated to be effective against different tumor cell lines, and to specifically target mononuclear phagocytes by activating the caspase 8 cascade via TRAIL receptors, which are expressed in monocytes and TAMs (Figure 3) [69,70]. We demonstrated that CD4 and CD8 T cells accumulated to a lesser extent in PDA compared to the normal pancreas, and highly produced IL10 but not IFNγ especially CD4 and Treg cells [71]. This was paralleled by the enrichment of H3K4me3, an active gene histone mark, at the promoter of Il10 in sorted tumor-infiltrated CD4 T cells and Tregs. Both cell types also showed a decreased level of H3K27me3, a repressive mark, at the promoter of T-bet [71]; T-bet being the main transcription factor that induces IFNγ expression in T cells [72]. When we depleted TAM by Trabectedin treatment, TIL CD4 cells displayed a higher production of IFNγ, and much less IL10, compared to the same population in untreated tumors (Figure 3). Again, this phenotype was confirmed by the epigenetic profile of sorted CD4 T cells, which showed a significant enrichment of the active mark H3K4me3 at the T-bet promoter and a decrease of H3K4me3 at the Il10 promoter [71]. In vitro analyses of generated macrophages treated with Trabectedin, or untreated, demonstrated that 17 out of 32 cytokines/chemokines were up-regulated by the treatment, while only CCL12 was down-modulated. Among the significantly up-regulated cytokines/chemokines were IL2, IL12, IL17 and TNFα, which are involved in T cell activation (Figure 3) [71]. Therefore, Trabectedin before inducing death of macrophages induces an increase in inflammatory cytokine and chemokine production, which shapes and regulates the epigenetic landscape of specific promoters related to the activation and phenotype of T cells. This effect renders Trabectedin, and the specific targeting of TAM, a suitable component for combinatorial therapies, which may open new effective ways to fight PDA. Further studies are ongoing to assess the efficacy of the combination of Trabectedin with ENO1 DNA vaccination in fighting PDA progression (Figure 4). Trabectedin, indeed, may lead to epitope spreading thanks to its cytotoxic effect on tumor cells, and the combined antigen-specific vaccination could enhance T cell reactivity.

Other new therapeutic strategies deploying epigenetic modulating agents also need to be considered for PDA. Some epigenetic drugs have been already tested in PDA with promising results, namely the inhibitor of histone methyltransferases, by Enhancer of Zeste Homolog (EZH) 2 or histone deacetylases (HDACs) [73], but no effects on immune infiltrating cells have been described.

## 4. Other Immunotherapy-Based Approaches in PDA Treatment

There are other types of immunotherapy currently being tested in clinical trials for PDA, which include whole cell, peptide, DNA transfected tumor cells, antigen pulsed-DC vaccines and monoclonal antibody treatments.

Whole cell vaccines typically use irradiated PDA cells as immunogens. These cells have the potential to elicit a robust immune response because they express the full repertoire of tumor-associated antigens. Algenpantucel-L is one of the most clinically advanced and promising immunotherapies; it is an irradiated, live combination of two human allogeneic PDA cell lines that express the murine enzyme α-1,3-galactosyl transferase (αGT), which directs the synthesis of α-galactosyl epitopes, usually absent in humans, and therefore has the potential to be strongly recognized by the immune system. Algenpantucel-L causes a hyperacute rejection of such allografts in humans, which is thought to trigger an immune response against cancer cells [74]. Another whole cell vaccine consists of irradiated tumor cells expressing the murine granulocyte-macrophage colony-stimulating factor (GM-CSF) named GVAX. This caused a potent, long-lasting antitumor response requiring both CD4^+^ and CD8^+^ T cells in the melanoma system [75]. The first peptide vaccine applied to PDA in a clinical trial was the synthetic Ras-peptide vaccine, which was proven to be safe and induce a good immune response in longer survivors [76,77]. Those promising results prompted the start of a clinical trial enrolling more than 100 patients, of which no results are available, unfortunately. Other peptide vaccines investigated in clinical trials with PDA patients include the telomerase peptide vaccine (GV1001) [78], the carcinoembryonic antigen (CEA), alone or in combination with mucin-1 (MUC-1). The CEA antigen has been triggered through a combination with a poxvirus-based vaccine containing three T-cell costimulatory molecules (TRICOM): B7-1 (CD80), intracellular adhesion molecule 1 (ICAM 1) and leukocyte function associated antigen-3 (LFA-3), while MUC-1 through a different viral-expressing vaccine (PANVAC-V, vaccinia virus, to immunize and PANCAV-F, fowl-pox virus, to boost) in combination with GM-CSF. No clinical benefits, however, were reported over canonical chemotherapy [79]. CEA and MUC-1 antigens were also used to pulse DC purified from patients and re-infused after in vitro expansion and loading. Both these DC-based vaccines were demonstrated to be safe, well-tolerated and elicited remarkable T cell responses [79,80]. MUC-1 mRNA-transfected autologous DC were used to vaccine unresectable or recurrent PDA patients in combination with gemcitabine and IL2 to expand cytotoxic T cells. The median survival appeared longer than that of patients receiving gemcitabine alone and only 5 out of 35 patients with no liver metastasis before treatment, did show metastasis after treatment [81]. Mesothelin is another interesting antigen that has been initially characterized in ovarian cancer and PDA [82]. CD8 T cell reactivity to mesothelin was described in PDA patients receiving GVAX and cyclophosphamide, either with or without live attenuated-Listeria monocytogene-expressing mesothelin [83]. Since 2014 at least five new clinical trials with mesothelin-chimeric antigen receptor (CAR) T cell adoptive therapy started (ClinicalTrials.gov identifier: NCT02159716, NCT01583686, NCT01897415, NCT02580747 and NCT02465983), as well as with CEA and MUC-1 CAR T cells (NCT02349724, NCT02416466 and NCT02587689 respectively). Phase I clinical trials were also performed with Wilms tumor (WT-1) peptide-based vaccine in combination with gemcitabine [84] and Cancer testis (CT) peptide-based vaccine in combination with vascular-endothelial growth factor receptor (VEGF-R1) and 2 (VEGF-R2) proteins, in which clinical benefits were observed even if in a trial with few patients [85].

Beside the immunotherapeutic strategies based on vaccinations, in recent years the potential role of immune checkpoint inhibitors in cancer treatment has become a field of great interest. The immune checkpoint molecules, such as CTLA-4 and PD-1, are expressed on the surface of activated T cells and their ligands, CD80/CD86 and PD-L1 respectively, are expressed mostly on APCs. The ligand-receptor interaction leads to the interruption of the inflammatory immune response and many tumors, including also PDA, express immune inhibitor molecules, such as PD-L1, to evade natural anti-tumor immunity [86]. The effectiveness of the use of checkpoint inhibitor to potentiate the anti-tumor T cell response and proliferation in several types of cancers has already been shown [87]. To date, inhibition of the PD-1/PD-L1 axis has produced impressive response rates in various malignancies, such as metastatic melanoma [88], renal [89] and non- small cell lung cancer (NSCLC) [90]. The tumor microenvironment of resected pancreatic cancer patients is rich in immune inhibitory molecules and the high expression of those molecules together with TILs is associated with better survival [86]. Despite some contrasting reports correlating PD-L1 expression with a poorer prognosis [91], the overall knowledge on the role of this pathway in PDA is still limited. Indeed, unlike the responses obtained in PD-1/PD-L1 clinical trials in other cancers, no objective responses were seen in a limited number of PDA patients with a single treatment [92]. Multiple PD-1 and PDL-1 inhibitors alone or in combination with chemotherapy are under investigation but without reported results to date (ClinicalTrials.gov identifier: NCT02988960, NCT02309177, NCT02331251, NCT02715531) [93]. Due to the poor success of single agent checkpoint inhibition, different approaches were integrated including dual checkpoint blockade and multi-modality immunotherapy or traditional therapy [93]. Combined immunotherapy strategies consisting in CTLA-4 blockade and GVAX, has already displayed benefit in a phase I study versus anti-CTLA-4 alone, with a median overall survival (OS) of 3.6 vs. 5.7 months and one year OS of 7% vs. 27% [94]. Considering the increased benefits of multi-combined therapy in comparison to single agent treatment in PDA, together with the promising preclinical results [95], immune checkpoints blockade could also be associated to TAAs vaccination. The observed anti-tumor immune activity elicited by ENO1 vaccination [17], indeed, could be potentiated and prolonged through the disruption of the tumor induced inhibitory brake (Figure 4).

## 5. ENO1 as Metabolic Target in Cancer Treatment

Recent evidence has shown that ENO1, in addition to its well characterized glycolytic functions, plays a role in pathophysiological processes; for example, by using an alternative stop codon, ENO1 can be translated into a 37kDa protein, named c-myc promoter-binding protein 1 (MIP1), which is a nuclear protein and able to bind the c-myc P2 promoter to negatively regulate transcription of this oncogene [96]. Although ENO1 is expressed in most of cells, its gene is not considered a housekeeping gene since its expression varies according to the pathophysiological, metabolic or developmental conditions of cells [97]. Specifically, ENO1 translation is upregulated during cellular growth, but barely detectable during the quiescent phase [98,99]. Indeed, numerous reports have shown an upregulation of ENO1 in several cancer types [100,101,102].

Knockdown of ENO1 in different tumor cell lines has led to a strong increase in their sensitivity to microtubule-targeted drugs (e.g., vincristine and taxanes), due to ENO1-tubuline interactions and also suggests a role for ENO1 in the microtubule network [103]. This effect seems related to the drastic reduction in invasiveness, e.g., in follicular thyroid carcinoma cells [104]. Likewise, ENO1 overexpression has been associated with poor clinical outcome in patients with head and neck cancer, and exogenous ENO1 expression promoted cell proliferation, migration, invasion and tumorigenesis [105]. Gene network analysis has also identified desmin, interleukin 8 and ENO1 as key elements for colon cancer tumorigenesis [106].

The role of ENO1 in PDA has been extensively documented, and ENO1 has been shown to promote cellular metabolism in anaerobic conditions, and drive tumor invasion through plasminogen activation and ECM degradation [107].

During tumor formation and expansion, tumor cells increase glucose metabolism [108]. Consistent with this, overexpression of glycolytic genes has been found in a variety of human cancers, including PDA [4,6,109]. ENO1 is one of the leading regulators of the Warburg effect and thus plays a major role in carcinogenesis and tumor maintenance [110]. ENO1 silencing in tumor cells decreased their proliferation and also affected in vivo tumor growth [110,111]. Interestingly, ENO1-silenced cells were able to resist glycolytic shutdown by rescuing oxidative phosphorylation. In the absence of ENO1, the decrease in lactate production and increase in ATP demand promoted glucose uptake and eventually led to the accumulation of intermediate glycolytic metabolites. Therefore, the excess of intracellular glucose was redistributed towards alternative pathways, such as the polyol pathway (PP) and the pentose phosphate pathway (PPP) to support cell growth and survival [110]. As demonstrated by the use of the PPP inhibitor, namely DHEA, NADPH oxidase hyper-activation was a consequence of the increased PPP flux and further contributed to the synthesis of superoxide. Moreover, reactive oxygen species (ROS) were responsible for the induction of senescence and growth arrest in ENO1-silenced cells [110,112]. We also observed that ENO1 silencing promoted catabolic pathway adaptation and fueled the TCA cycle by anaplerotic reactions of tyrosine and glutamine catabolism, another important molecule for PDA metabolic adaptation [113].

The above considerations led to the hypothesis of targeting ENO1 to simultaneously disturb cancer cells in multiple ways. An interesting report by Jung et al. described a different cell permeable glycolysis inhibitor (AP-III-a4) able to bind the outer active site of ENO1 hence dubbed “ENOblock” [114,115]. However, it was reported that ENOblock is not able to inhibit ENO1 activity in vitro [116]. Fortunately, there are four compounds classified as non-mutagenic and non-carcinogenic, with a steady interaction with ENO1 that were comparable, or even superior, to the currently available inhibitors: AEP, PhAH, and SF-2312. These compounds, namely ZINC1304634, ZINC16124623, ZINC1702762, and ZINC72415103, may be considered promising for further development of ENO1 inhibitors, and could help fight cancer metabolically [117].

Given the complex metabolic switch with variable changes in expression of enzymes in pancreatic cancer, altering expression levels of ENO1 with metabolic inhibitors has shown an encouraging effect [118]. To date, there are no clinical trials involving metabolic inhibitors in PDA. However, there has been good progress in using metabolic inhibitors in cell types other than PDA, which have proven to have good translationability [119,120].

## 6. Conclusions

Having established that immunological targeting of ENO1 by DNA vaccination is a powerful stimulus for humoral and cellular responses against PDA (Figure 1 and Figure 4), the next generation of immunotherapy will take advantage of recent data on the effects of chemotherapy to extend and amplify the immune response against ENO1 and predispose the immune system to promptly respond to ENO1 and other TAAs, as well as data demonstrating the effectiveness of the inhibition of PI3K isoforms to unleash antitumor responses in PDA. In addition, Trabectedin has proven to be effective in depleting tumor-associated macrophages that infiltrate PDA and epigenetic reprograming TILs into antitumor effector cells. Finally, ENO1 inhibition may contribute to reducing the proliferative and invasive ability of PDA cells and to inducing their senescence. As all these approaches utilize drugs or compounds that are used or already approved for clinical purposes, they represent an evaluable springboard for developing—in a short time—a more efficacious protocol for the next generation of PDA immunotherapy based on DNA vaccination (Figure 4).
